# Genomic regions influencing intramuscular fat in divergently selected rabbit lines

**DOI:** 10.1111/age.12873

**Published:** 2019-11-07

**Authors:** Bolívar S. Sosa‐Madrid, Pilar Hernández, Agustín Blasco, Chris S. Haley, Luca Fontanesi, María A. Santacreu, Romi N. Pena, Pau Navarro, Noelia Ibáñez‐Escriche

**Affiliations:** ^1^ Institute for Animal Science and Technology Universitat Politècnica de València 46022 Valencia Spain; ^2^ MRC Human Genetics Unit MRC Institute of Genetics and Molecular Medicine University of Edinburgh Crewe Road, Edinburgh EH4 2XU United Kingdom; ^3^ Roslin Institute and Royal (Dick) School of Veterinary Studies University of Edinburgh Midlothian EH25 9RG United Kingdom; ^4^ Division of Animal Sciences Department of Agricultural and Food Sciences University of Bologna 40127 Bologna Italy; ^5^ Departament de Ciència Animal Universitat de Lleida–Agrotecnio Centre E-25198 Lleida Catalonia, Spain

**Keywords:** divergent selection, genome‐wide association study, intramuscular fat, meat quality, rabbits

## Abstract

Intramuscular fat (IMF) is one of the main meat quality traits for breeding programmes in livestock species. The main objective of this study was to identify genomic regions associated with IMF content comparing two rabbit populations divergently selected for this trait, and to generate a list of putative candidate genes. Animals were genotyped using the Affymetrix Axiom OrcunSNP Array (200k). After quality control, the data involved 477 animals and 93 540 SNPs. Two methods were used in this research: single marker regressions with the data adjusted by genomic relatedness, and a Bayesian multiple marker regression. Associated genomic regions were located on the rabbit chromosomes (OCU) OCU1, OCU8 and OCU13. The highest value for the percentage of the genomic variance explained by a genomic region was found in two consecutive genomic windows on OCU8 (7.34%). Genes in the associated regions of OCU1 and OCU8 presented biological functions related to the control of adipose cell function, lipid binding, transportation and localisation (*APOLD1*,* PLBD1*, *PDE6H*, *GPRC5D* and *GPRC5A*) and lipid metabolic processes (*MTMR2*). The *EWSR1* gene, underlying the OCU13 region, is linked to the development of brown adipocytes. The findings suggest that there is a large component of polygenic effect behind the differences in IMF content in these two lines, as the variance explained by most of the windows was low. The genomic regions of OCU1, OCU8 and OCU13 revealed novel candidate genes. Further studies would be needed to validate the associations and explore their possible application in selection programmes.

## I**ntroduction**

Intramuscular fat (IMF) contributes to improve organoleptic properties and sensory attributes of the meat, as demanded by consumers (Hocquette *et al.* 2010). Hence, a large number of studies have investigated the genetic factors controlling IMF content in meat and their implications for several species, e.g. in beef cattle (Sapp *et al.* 2002; Garrick 2011; Ochsner *et al.* 2017), swine (McLaren & Schultz 1992; Gao *et al.* 2007), sheep (Hopkins *et al.* 2011; Mortimer *et al.* 2014) and goats (Peña *et al.* 2011). Following these studies, IMF has emerged as one of the most important meat quality parameters and in a few cases it has been included in breeding programmes (Gotoh *et al.* 2018; Pannier *et al.* 2018).

Moderate‐to‐high heritability and large variability have been reported for livestock IMF traits, which argue for a good potential for improving meat quality through genetic selection. IMF heritability is around 0.53 in swine (Ros‐Freixedes *et al.* 2016), 0.38 in cattle (Mateescu *et al.* 2015), 0.48 in sheep (Mortimer *et al.* 2014) and 0.54 in rabbit (Martínez‐Álvaro *et al.* 2016). Important limitations to IMF selection are the IMF being recorded mainly at slaughter and the phenotyping process being costly. In this context, genetic marker selection based on quantitative trait locus (QTL) with high or moderate effect size could overcome some of these limitations.

At genomic level, studies carried out in beef cattle suggest that IMF could be influenced by a large number of genes (Strucken *et al.* 2017). Nevertheless, studies in Japanese Black cattle have reported genomic markers with large effects on IMF or marbling score around the *SCD*, *FASN*,* AKIRIN2*,* EDG1 *and* RPL27A* genes (Gotoh *et al.* 2014; Sukegawa *et al.* 2014). Genomic markers on the genes *SCD* and *FASN* have been incorporated into a breeding programme for this breed to select elite sires (Gotoh *et al.* 2018). In swine, similarly to beef cattle, the results of experiments associating genetic markers with IMF are hardly conclusive with regard to the magnitude and importance of discovered associations (Pena *et al.* 2016). However, traits correlated to IMF such as fatty acid profiles have shown a noteworthy QTL on chromosome 14 in a Duroc commercial line (Uemoto *et al.* 2012; Ros‐Freixedes *et al.* 2016). So far, IMF appears as a troublesome trait for mapping studies in livestock species, owing to either the lack of validation in the results or insufficient power to detect genetic causal variants. Thus, genomic studies to understand the genetic control of IMF are still needed.

The rabbit has been shown to be an excellent animal model for other livestock species (Miller *et al.* 2014). Further, the recent availability of a high‐density SNP array has facilitated the performance of genomic studies. At the Universitat Politècnica de València, a successful divergent selection experiment for IMF has been carried out (Martínez‐Álvaro *et al.* 2016). The developed rabbit lines were kept in the same environment and selection criteria only differ for the IMF selection objective. Selection could have modified SNP frequencies in opposite directions, leading to intermediate allelic frequencies when both lines are jointly considered. This could increase the detection power of associated loci in a genome‐wide association study (GWAS) based on this experimental design.

The aim of this study was to carry out GWASs using these divergently selected rabbit lines to identify genomic regions associated with IMF and generate a list of putative candidate genes affecting this trait. Two different methods (single marker regression, SMR, and Bayesian multiple marker regression, BMMR) were applied to confirm the identified relevant genomic regions.

## Materials and methods

### Ethical statement

All experimental procedures were approved by the Ethical Committee of the Universitat Politècnica de València, according to Council Directives 98/58/EC (European Economic Community, 1998).

### Animals and phenotypes

The animals of this study came from two rabbit lines divergently selected for IMF during nine generations at the Universitat Politècnica de València. The base population was composed of 83 does and 13 males from a synthetic rabbit line (Zomeño *et al.* 2013). The selection criterion was IMF content collected in two full siblings of the first parity. The selection of the males was within the sire family, avoiding mating between cousins to control inbreeding. At the ninth generation, the high‐IMF line consisted of 55 does and 10 males, and the low‐IMF line consisted of 61 does and 10 males. Over all animals, the mean was 1.09 g of IMF per 100 g of *Longissimus thoracis et lumborum* (LTH) muscle, after adjusting data for systematic effects (parity order, line, month‐season and sex) and a common litter random effect. The high‐IMF line had a mean of 1.27 g/100 g of LTH with 0.21 standard deviations, and the low‐IMF line had a mean of 0.83 g/100 g of LTH with 0.07 standard deviations. Details about the IMF divergent selection experiment can be found in Martínez‐Álvaro *et al. *(2016). The selection response was around 3.1 standard deviations at the ninth generation, calculated as the difference between lines. The phenotypic difference between lines was 41% of the mean of the base population.

The rabbits were brought up jointly from 33 days at weaning until slaughter under the same handling and feeding conditions. At 9 weeks from birth, the rabbits were slaughtered following a fasting period of 4 h. Carcasses were chilled 24 h at 2.5 °C after slaughter and dissected to obtain a sample of the left LTH muscle for each animal. These samples were minced, frozen, lyophilised and milled. The IMF data were obtained using near‐infrared spectroscopy (model 5000; FOSS NIRSystems Inc., Hilleroed, Denmark; Zomeño *et al.* 2013; Martínez‐Álvaro *et al.* 2016). In the last generation, 729 samples of the left LTH muscle of each animal were collected and IMF measured to compute the IMF selection response, and 480 rabbits were chosen from groups of an average size of four siblings per doe (dam) for the GWAS.

### Genotyping and quality control

*Obliquus abdominis* muscle specimens (~50 g), obtained after slaughter of the animals, were used for DNA extraction using a standard protocol (Green *et al.* 2012). A total of 480 individuals were genotyped using the Affymetrix Axiom OrcunSNP Array (Affymetrix Inc., Santa Clara, CA, USA) at the ‘Centro Nacional de Genotipado’ (CeGen), Universidad de Santiago de Compostela. The SNP array contains 199 692 genetic molecular markers. The quality control was performed using axiom analysis suite version 3.0.1.4 and zanardi (Marras *et al.* 2017). SNPs with a call rate of at least 0.95, MAF of at least 0.03 and a known autosomal chromosome position according to OryCun2.0 assembly (Carneiro *et al.* 2014) were used in the analyses. Furthermore, animals missing more than 3% of marker genotypes, or failing a Mendelian inheritance test, were excluded. The remaining missing genotypes were imputed by the software beagle version 4.0 (Browning & Browning 2016). The SNPs with an imputation quality score *R* ^2^> 0.75 were included. After filtering, the data included 477 animals (240 from the high‐IMF line and 237 from the low‐IMF line) and 93 540 SNPs. In addition, the SNP density was described in this research because the rabbit SNP array is new (Blasco & Pena 2018).

### Genome‐wide association study

Prior to performing the GWAS, we performed a multidimensional scaling analysis to evaluate the population structure in our genomic data. The method treats the distances as Euclidean distances and preserves the original distance metric, between points, as well as possible (Borg & Groenen 2005). The command cmdscale() from the R package *stats* was used to implement this analysis (R Core Team 2013).

Two methods were employed in this study: a frequentist and a Bayesian. Both methods included the mean and the systematic effects in the model: month‐season (five levels), sex (two levels), order‐parity (three levels) and line (two levels). The inclusion of a common litter random effect in the model was evaluated owing to the importance of this effect in previous studies of IMF in rabbits (Martínez‐Álvaro *et al.* 2016). Inclusion of this effect did not affect GWAS results (not shown), hence for simplicity we excluded this effect in the GWAS.

*Single marker regression (SMR) with the data adjusted by genomic relatedness.* The analysis was implemented using a family‐based score test for association (FASTA). The SNP effects were evaluated with FASTA based on a polygenic‐lineal mixed model that included the genomic kinship matrix to explain relatedness in the sampled population (Chen & Abecasis 2007). The model equation was:

y=1μ+Xb+βg+Zu+e

where y is the vector of IMF phenotypes, 1 is a vector of ones, μ is the trait mean, X is the design matrix for the systematic effects, b is the vector of systematic effects, β is the substitution effect for a particular SNP, g is the vector of genotypes for each SNP denoted as the number of reference alleles for a particular SNP (0, 1 or 2), Z is the design matrix for random polygenetic effects, u is the vector of random polygenic effects with a normal distribution $N(0,{G∙\sigma }_{u}^{2})$ and e is the vector of random residual effects with a normal distribution$N(0,{I∙\sigma }_{e}^{2})$; $\sigma_{u}^{2}$ is the genomic variance and G is the genomic kinship matrix computed using the genomic data by the method of Astle & Balding (2009). The identity matrix was denoted as I and $\sigma_{e}^{2}$ is the residual variance. The implementation of the association analysis was performed using R software package genABEL (Aulchenko *et al.* 2007). Furthermore, we utilised a genomic control method to avoid inflation in the statistic test. We calculated the lambda parameter that indicates the excess of false positives in the results. When its application is needed, the regression factor *λ* corrects the observed *P*‐values leading to new *P*‐values for every assessed SNP (Aulchenko *et al.* 2007). In this research, we used two thresholds: an LD‐adjusted Bonferroni (8.12 × 10^−6^) calculated for 10 Mb LD blocks according to LD analysis implemented in plink (Purcell *et al.* 2007), and also, a suggestive threshold of 1 × 10^−4^ owing to the high relatedness of the samples (Lander & Kruglyak 1995; Sahana *et al.* 2011; Do *et al.* 2018). As Bonferroni is a conservative method, we also implemented the suggestive threshold because it is less stringent as the samples from animals with high relatedness would have genomic segments of LD larger than those in humans (Wang *et al.* 2016c; Schmid & Bennewitz 2017). Therefore, the number of independent sites could be overestimated causing false‐negative results if SNP density is not large enough to adjust Bonferroni by LD (Spencer *et al.* 2009; Do *et al.* 2014).

*Bayesian multiple marker regression (BMMR).* This method is more robust to population structure than SMR approaches (Toosi *et al.* 2018). However, the line effect would correct for potential biases that might be derived by the family‐data structures in the investigated rabbit populations. Thus, the line effect remained in the BMMR model. The parameters were estimated with the following Bayes B model (Cesar *et al.* 2014; Ros‐Freixedes *et al.* 2016):

$$y=1\mu +Xb+\sum_{j=1}^{k}z_{j}\alpha_{j}\delta_{j}+e$$

where y, 1, X, b and e are the same as in the frequentist method shown above, $z_{j}$ is the vector including the genotypic covariate for each SNP or locus j (0, 1 or 2), $\alpha_{j}$ is the random substitution effect for SNPj and $\delta_{j}$ is the random 0/1 variable that represents the presence ($\delta_{j}$ = 1 with probability 1 − *π*) or absence ($\delta_{j}$ = 0 with probability *π*) of SNPs in the model for a given iteration. The value of *π* is defined as the proportion of SNPs with zero effects in the model. The value of *π* in our study was 0.9988, which means that between 100 and 200 SNP markers have non‐zero effects for every iteration. The parameters of the model were estimated with marginal posterior distributions using Markov chain Monte Carlo. After some exploratory analysis, a total of 825 000 iterations were performed, with a burn‐in period of 225 000 iterations. Only one sample every 60 iterations was saved to avoid the high correlation between consecutive samples. Gensel ^®^ version 4.90 software (Garrick & Fernando 2013) was used for the GWAS analysis. The relevance of the association was assessed using two criteria, the Bayes factor (Stephens & Balding 2009; Ros‐Freixedes *et al.* 2016) and the percentage of the genomic variance explained for non‐overlapping genomic windows of 1 Mb, calculated by marginal posterior density. The genomic windows were defined for each chromosome and according to the OryCun2.0 rabbit genome assembly (Carneiro *et al.* 2014). In our study, 1999 genomic windows were defined. Those windows accounting for at least 1.0% of the total genomic variance were considerate as important to continue with the subsequent analysis (Cesar *et al.* 2014). This threshold was 20 times greater than the average genomic variance explained by a window (0.05%). We also considered the consecutive windows that explained at least 0.5% of genomic variance having a strong LD between them (Ros‐Freixedes *et al.* 2016) as SNPs associated with a causal variant can be located between consecutive windows and the estimated effect of association could be divided among these windows, hindering the detection of a genomic region (Beissinger *et al.* 2015).

In this study, we integrated the results from both frequentist and Bayesian methods to define the relevance of associations. This was established by the following procedure: first, we drew all genomic windows that overcame the condition expressed in the above paragraph. Then, the genomic windows harbouring SNPs above or around a Bayes factor of 20 (Kass & Raftery 1995) were extracted and considered as relevant genomic windows. These SNPs reaching at least one of thresholds, either suggestive or Bayes factor thresholds, were denoted as relevant polymorphisms. Finally, the genomic regions having relevant associations were chosen for functional gene analysis.

In addition, the three main important polymorphisms within relevant genomic regions were tested according to genotypes using contrasts by frequentist statistic. This test was carried out within the IMF line in order to evaluate the statistical differences amongst genotypes of SNPs. To do that, a general linear model was implemented using R software (R Core Team 2013).

### Linkage disequilibrium and functional gene analysis

To evaluate the number of independent sites across the rabbit genome, a computation of LD for blocks was performed. The plink software was utilised to identify LD blocks (Purcell *et al.* 2007). The number of independent sites was calculated every 0.5, 1, 5, 10 and 20 Mb (genomic physical distance) across the whole rabbit genome. The LD‐adjusted Bonferroni threshold used in this study was calculated using the number of independent sites for 10 Mb as the number of independent sites barely changed between 10 and 20 Mb. LD blocks were examined in the associated genomic regions through the Haploview software (Barrett *et al.* 2005). In order to visualise the genes into the relevant genomic regions (±500 kb of associated SNP), we initially used the programme UCSC Genome Browser (https://genome.ucsc.edu/cgi-bin/hgGateway). The gene annotations were determined using Ensembl Genes 96 Database in biomart (Aken *et al.* 2016). The functional enrichment and metabolic pathways analysis were finally performed using the Database for Annotation, Visualization and Integrated Discovery (david) version 6.8 (Jiao *et al.* 2012) and enrichr (Kuleshov *et al.* 2016). The computation for the functional analyses was carried out using the parameters recommended by the authors. In addition, the search for annotated functions for each gene was performed individually using the database of all annotated functions from Ensembl and david.

## Results

### Genomic data

A total of 93 540 autosomal SNPs with known chromosomal positions were retained after filtering for MAF and call rate (see details in Materials and Methods). The number of retained SNPs on each of the 21 rabbit autosomes is shown in Table 1. The average physical distance between these SNPs was 22.61 kb. The average SNP number within 1 Mb windows was 46. One extended genomic region on OCU14 (54–65 Mb) did not contain any SNPs.

**Table 1:** Allocation of SNPs after quality control and average distance amongst contiguous SNPs on every chromosome.

OCU	Number of SNPs	Percentage of SNPs in OCU^1^	Average distance (kb)	Chromosome size (Mb)
1	9288	63	20.98	194.85
2	7856	58	22.19	174.33
3	7006	59	22.22	155.69
4	3895	58	23.47	91.39
5	1721	67	21.84	37.99
6	1222	63	22.48	27.50
7	7626	57	22.78	176.68
8	5075	57	22.03	111.80
9	5136	57	22.58	116.25
10	2318	61	19.38	48.00
11	3827	56	22.81	87.55
12	7116	60	21.83	155.35
13	5945	56	24.11	143.36
14	5687	45	28.81	163.90
15	4657	55	22.71	109.05
16	3962	62	21.32	84.48
17	3836	59	21.94	85.01
18	3102	64	21.45	69.80
19	2574	64	21.00	57.28
20	1224	51	24.66	33.19
21	467	55	26.56	15.58
Total	93 540	47		

^1^ The proportion of SNPs after quality control divided by number total of SNPs into OCU (rabbit chromosome) from the rabbit SNP array.

### GWAS for IMF

**Figure 1:**
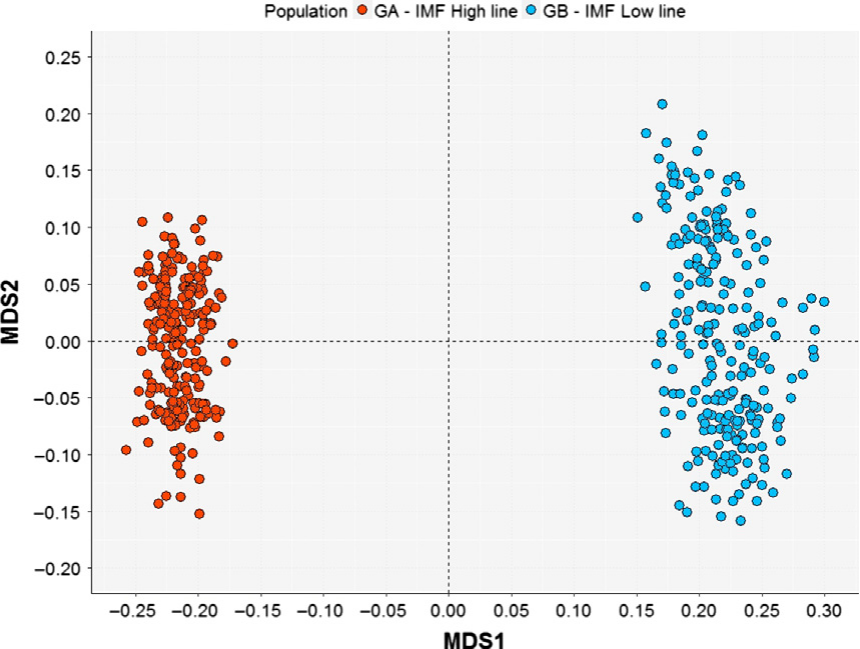
Multidimensional scaling plot of genomic data. The first component (MDS1) explained 29.26% of the genomic variance and the second component (MDS2) explained 3.26% of the genomic variance.

Figure 1 reports a multidimensional scaling plot obtained using the genotyped SNPs on the rabbits of the two divergent IMF lines. A strong structure separating the high‐ and low‐IMF lines is evident. Therefore, a line effect was included in the models. In addition, a polygenic effect was also included in the SMR to adjust this model owing to the plausible effects derived from family‐data structures, considering a genomic kinship matrix. After this correction, the calculated lambda parameter was 1.065, indicating that the correction of bias derived from the population structure was not enough. Hence, we also implemented the correction by the lambda parameter in the SMR analysis. Note that the first and second components of multidimensional scaling accounted for 29.26% and 3.26% of genomic variance, respectively (Fig. 1).

**Figure 2:**
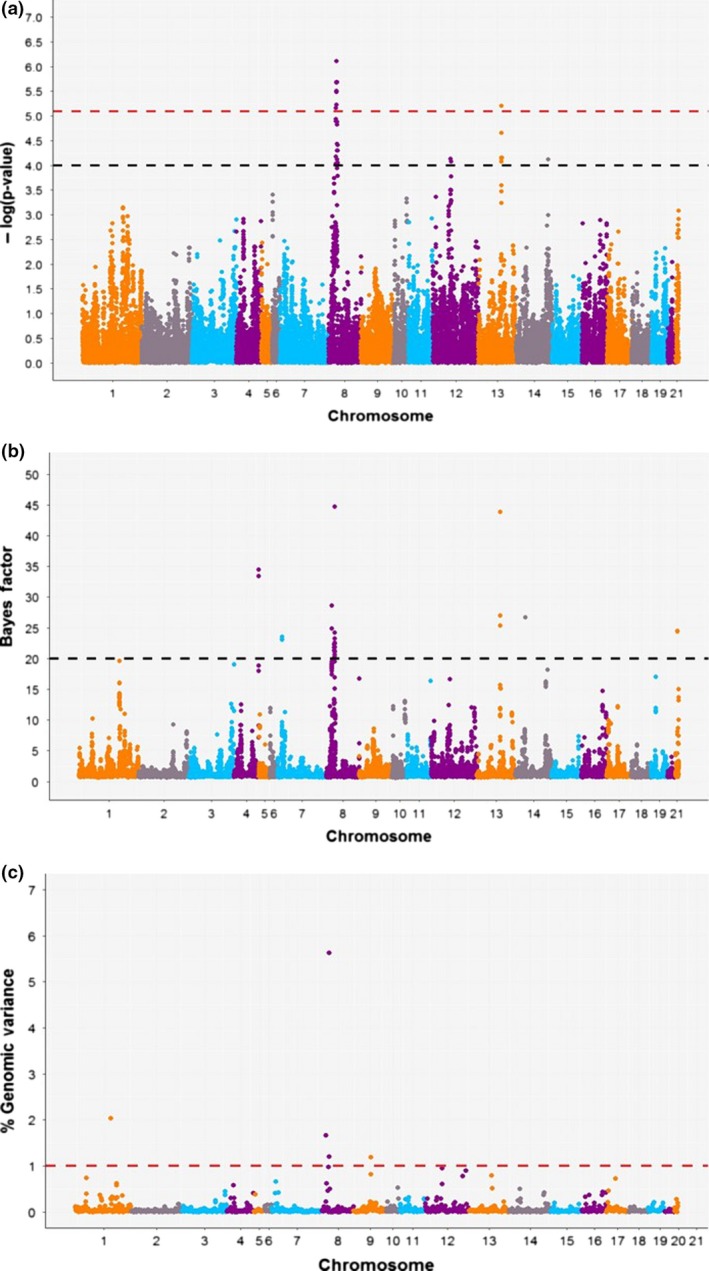
Manhattan plot for each model. (a) Single marker regression adjusted by genomic relationship. The − log (*P*‐value) thresholds are 5.09 (LD‐Bonferroni – red dashed line) and 4.0 (suggestive – black dashed line). (b) The Bayes factor for each SNP for the Bayesian multimarker regression model. The black dashed line indicates the Bayes factor threshold of 20. (c) The percentage genomic variance explained by each non‐overlapping 1 Mb window for the Bayesian multimarker regression model (threshold of 1% – red dashed line).

Two methods were used in this research: SMR with the data adjusted by genomic relatedness and a BMMR (Bayes B method). We employed the term of "relevant" in order to denote those SNPs and genomic windows that we considered as true positive associations. In this research, we understand the GWAS as an exploratory analysis, which works as a mechanism for deriving promising genomic regions associated with IMF, and retrieving annotated rabbit genes. Table 2 shows the SNPs and genomic windows associated with IMF according to the procedure for defining the relevant associations (see details in Materials and Methods). For both methods, the associated SNPs and genomic windows were located on OCU8 and OCU13. The two genomic windows on OCU13 (2 Mb), containing  10 relevant SNPs for both methods, accounted together for 1.30% of the total genomic variance. On OCU8, 10 relevant polymorphisms showed the lowest *P*‐values for the SMR method, and had high Bayes factors for the BMMR method (Fig. 2). The two genomic windows containing these relevant polymorphisms accounted for 7.34% of the genomic variance. In addition, a genomic window on OCU1 was found to be associated with IMF by BMMR, explaining 2.03% of the genomic variance. The associated SNPs in this latter genomic window presented values close to the Bayes factor threshold, but these SNPs were distant from the *P*‐value (suggestive) threshold for SMR method.

**Table 2:** Relevant polymorphisms (SNPs) and genomic windows associated with intramuscular fat.

SNP name	OCU	Position (bp)	*P*‐Value	Bayes factor	Window		MAF
					Name	Percentage of variance	
Affx‐151793092	1	121151928	1.10 × 10^−3^	15.95	118	2.03	0.24
Affx‐151803947	1	121280205	1.10 × 10^−3^	19.59			0.24
Affx‐151888965	1	121308004	1.10 × 10^−3^	16.03			0.25
Affx‐151956200	8	14893810	3.51 × 10^−4^	19.51	831	1.21	0.31
Affx‐151962168	8	14913105	3.51 × 10^−4^	24.86			0.32
Affx‐151945237	8	14939285	3.51 × 10^−4^	28.58			0.31
Affx‐151973204	8	14972879	1.83 × 10^−4^	18.38			0.31
Affx‐151800097	8	25087426	2.13 × 10^−6^	21.78	841	6.20	0.16
Affx‐151900210	8	25227502	3.33 × 10^−6^	44.73			0.16
Affx‐151917268	8	25262821	2.13 × 10^−6^	20.64			0.16
Affx‐151813008	8	25268392	2.13 × 10^−6^	22.57			0.16
Affx‐151795704	8	25467177	3.12 × 10^−6^	20.99			0.16
Affx‐151972842	8	25643667	2.06 × 10^−6^	24.15			0.16
Affx‐151964185	8	25732369	2.06 × 10^−6^	21.78			0.16
Affx‐152000638	8	25751303	2.06 × 10^−6^	21.17			0.16
Affx‐151808634	8	25863739	2.06 × 10^−6^	23.27			0.16
Affx‐151853378	8	25874631	2.12 × 10^−6^	21.25			0.16
Affx‐151824236	8	26115758	2.66 × 10^−3^	21.87	842	1.14	0.16
Affx‐151867012	13	84307591	7.14 × 10^−5^	11.73	1380	0.79	0.09
Affx‐151824373	13	84431723	7.14 × 10^−5^	10.62			0.09
Affx‐151874466	13	84447172	8.45 × 10^−5^	11.90			0.09
Affx‐151883028	13	84453332	7.14 × 10^−5^	11.73			0.09
Affx‐151801561	13	84537466	7.14 × 10^−5^	25.39			0.09
Affx‐151841215	13	84723427	2.20 × 10^−5^	25.39			0.09
Affx‐151846540	13	84738337	2.20 × 10^−5^	26.98			0.09
Affx‐151790364	13	84751504	2.23 × 10^−5^	25.30			0.09
Affx‐151939801	13	85316544	3.40 × 10^−4^	43.81	1381	0.51	0.08
Affx‐151937959	13	85333053	6.31 × 10^−6^	15.69			0.09

Percentage of variance: percentage of genomic variance explained by window. OCU, rabbit chromosome; bp, base pair.

**Figure 3:**
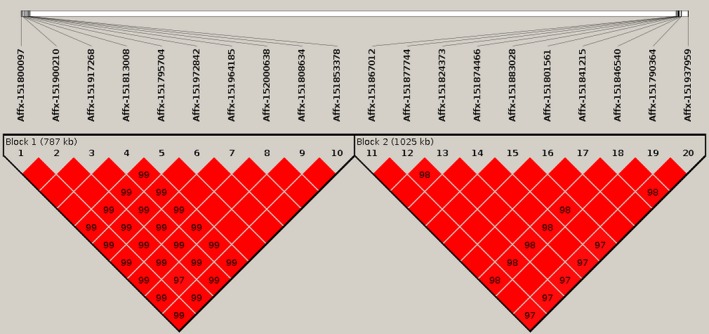
LD blocks from main relevant associated polymorphisms. Block 1 includes SNPs 1–10 on chromosome 8 in 24.59–26.95 Mb and block 2 includes SNPs 11–20 on chromosome 13 in 83.81–86.00 Mb.

Regarding the LD analysis, we found that in our data the rabbit genome could be divided into 2338 LD blocks and 6158 independent sites, with the longest LD blocks having a maximum length of 10 Mb. The associated SNPs on OCU13 and on OCU8 displayed a high LD within the chromosomal region (Fig. 3). The associated genomic region on OCU13 (window 1380 and 1381) holds two LD blocks. The second LD block (of 1506 kb) included almost all of the two associated windows (Fig. S1). The associated genomic region on OCU8 (window 841 and 842) presented just one block of 1945 kb, containing both windows (Fig. S2).

After the previous analysis (GWAS and LD), four relevant genomic regions were used to continue searching for putative candidate genes based on the functional annotation analysis (Table 3). In these regions, we also tested the IMF differences between genotypes within lines. Most of the SNPs tested presented statistical differences between one of the homozygous genotypes and the other genotypes within the high‐IMF line. In the low‐IMF line, except in region located 14.01–15.47 Mb in OCU8, these SNPs were not segregating (Fig. S3).

**Table 3:** Summary of relevant genomic regions associated with intramuscular fat and annotated rabbit genes.

Cluster	OCU	Position (bp)		Number of genes	Annotated rabbit gene
		Start	End		
1	1	120,651,928	121,986,803	9	*MAML2*,* MTMR2*,* CEP57*,* FAM76B*,* ENSOCUG00000025632* 1,* SESN3*,* ENDOD1*,* KDM4D*,* CWC15*
2	8	14,014,437	15,472,879	9	*RASSF8*,* LMNTD1*,* RF00001*,* KRAS*,* ETFRF1*,* CASC1*,* LRMP*,* BCAT1*,* ENSOCUG00000021067* 1
3	8	24,587,426	26,948,204	25	*PDE6H*,* ARHGDIB*,* ERP27*,* MGP*,* ART4*,* SMCO3*,* ENSOCUG00000017177* 1,* H2AFJ*,* HIST4H4*,* GUCY2C*,* PLBD1*,* ATF7IP*,* ENSOCUG00000017095* 1,* ENSOCUG00000021765* 1,* GRIN2B*,* RF00411*,* ENSOCUG00000021882* 1,* EMP1*,* GSG1*,* FAM234B*,* HEBP1*,* GPRC5D*,* GPRC5A*,* DDX47*,* APOLD1*
4	13	83,807,591	85,998,108	3	*RF00026*,* ENSOCUG00000027270* 1,* RF00001*

CLUSTER, denotes the genomic region; OCU, rabbit chromosome; bp, base pair.

^1^ Novel genes are named according to their Ensembl gene ID.

### Functional annotation analysis and putative candidate genes

The final objective of our study was to generate a list of putative candidate genes, in order to guide further research for investigating the genetic determination of IMF content. Overall, 46 genes were annotated to the four relevant genomic regions (Table S1).

Only three genes (two non‐coding‐protein genes and one protein‐coding gene) mapped to the genomic region on OCU13 (Table 3). Among them stands out a novel annotated gene with Ensembl gene ID: ENSOCUG00000027270 (84.56 Mb), which is linked to metal ion binding in rabbits. The genes located on the genomic region on OCU8 were those showing a clearer relationship to lipid metabolism pathways. The ‘apolipoprotein L domain containing 1’ gene (*APOLD1*) shows functions related to lipid binding, transportation and localisation. The ‘phospholipase B domain containing 1’ (*PLBD1*) and ‘phosphodiesterase 6H’ (*PDE6H*) genes show functions linked to hydrolase activity (phospholipases) and lipid metabolic processes. In humans, several functional annotations, including the sphingolipid signalling pathway, have been found for the ‘K‐RAS proto‐oncogene, GTPase’ (*KRAS*) gene. Moreover, two members of the retinol‐induced G protein‐coupled protein receptors also stand out in OCU8: ‘G protein‐coupled receptor class C group 5 member D’ (*GPRC5D*) and ‘G protein‐coupled receptor class C group 5 member A’ (*GPRC5A*; Table 3). On OCU1, the ‘myotubularin‐related protein 2’ (*MTMR2*) gene displays biological functions linked to lipid metabolic processes. In addition to the biological and molecular functional annotations, a list of pathways that include these genes was generated from david, the KEGG and Wiki pathways databases (Table S2).

## Discussion

Knowledge and understanding of control mechanisms of IMF content would be useful in the meat industry. Thus, a GWAS was performed in order to identify genomic regions associated with IMF content in rabbits owing to the increasing importance of meat quality in livestock for consumers (Hocquette *et al.* 2010; Pena *et al.* 2016; Strucken *et al.* 2017).

Following GWAS detection power studies (Spencer *et al.* 2009; Visscher *et al.* 2017), the distribution of SNPs (after quality control) across the rabbit genome in our data was suitable for GWAS analysis in livestock, given the LD and SNP density (Fan *et al.* 2010; Zhang *et al.* 2012). For instance, LD blocks having distance of 98 kb show *r* ^2^ = 0.5 as a measure of LD within rabbit breeds (Carneiro *et al.* 2011). This would indicate that the 93 540 SNP having an average distance of 22.61 kb between SNPs can be useful for discovering true associations amongst SNPs and the causal variants of IMF.

A challenge in GWAS analysis is the impact of confounding factors in the results. To avoid problems owing to population structure, we fit the genomic kinship matrix (Sul *et al.* 2018). The obtained *λ* value of 1.065 shows that this was almost enough to correct the population stratification effect. The purpose of implementing two methods was to corroborate the presence of associations between genomic windows or SNPs with IMF. The causal variants of moderate to high effect size can be detected by both methods in GWAS analyses when polymorphisms present high LD with these causal variants (López de Maturana *et al.* 2014). SNPs on OCU13 and OCU8 were found to be associated with IMF for both frequentist and Bayesian methods. However, the two associated windows on OCU13 (window 1380 and 1381) explained the low percentage of genomic variance (<1%). In addition, the LD block containing the most important SNPs on OCU13 covered a short physical distance and was uneven with regard to LD within this block (Fig. S1). This indicates that in this area of the genome a selective sweep process might not have been produced by divergent selection, since short‐term selection increases LD and the expected length of the LD block that contains an important causal variant (Vitti *et al.* 2013). In addition, the reference alleles of these associated SNPs presented low allelic frequencies (close to zero) for the low‐IMF line. The MAF value of the reference SNPs was also low (<0.09) in both low‐ and high‐IMF lines (Table 2). All SNPs were fixed or near fixation in the low‐IMF line, therefore the associations of these SNPs with IMF were uncovered given their segregation in the high‐IMF line. This could affect the association detection power even when the sample size is large (López de Maturana *et al.* 2014). For instance, if SNPs associated with the causal variants present a low MAF, the effects and association can be underestimated, generating false‐negative results.

In contrast, the associated region on OCU8 in 24.59–26.95 Mb explained a larger percentage of genomic variance between both associated windows (7.34%). Moreover, this region presented a strong and long LD block between windows 841 and 842, which could imply a selective sweep process owing to divergent selection (Fig. S2). The MAF values of the SNPs in this region were higher than on OCU13, reaching a maximum value of 0.16 (Table 2). Most SNPs in OCU8 were fixed or near fixation in the low‐IMF line. It seem that the causative variants and their surrounding SNPs would be at low frequency in the base population. This might explain the fixation of SNPs in the low‐IMF line and their segregation in the high‐IMF line of the ninth generation. Therefore, this genomic region showed more evidence than the region on OCU13 for considering it as an important association driving the control mechanism for IMF. Finally, another potentially interesting genomic region was identified on OCU1. This region explained 2.03% of the IMF genomic variance, although the SNPs show − log (*P*‐values) or Bayes factors below thresholds (Fig. 2). This suggests that the association of these SNPs could be better captured by a method that considers the percentage of variance explained by the windows instead of evaluating each SNP individually. In addition, these SNPs present MAF values around 0.24 (0.48 for the high‐IMF line and close to zero for the low‐IMF line), which might suggest that the differences might be a consequence of the divergent selection process.

This is the first GWAS study for IMF in rabbits. Therefore, comparisons within rabbits are limited to previous candidate gene studies. In this sense, as in Migdał *et al. *(2018), we did not find an association between the *FABP4* (OCU3) candidate gene and IMF. Our results are not in agreement with the studies for *FTO* (OCU5) (Zhang *et al.* 2013), *CAST* (OCU11) (Wang *et al.* 2016b) and *MYPN* (OCU18) (Wang *et al.* 2017), which found associations in two, one and one SNP within genes, respectively (*P*‐values between 0.032 and 0.044). However, these associations should be taken with caution as the significance threshold was more liberal (*P*‐value < 0.05, without applying correction for multiple testing) than in our GWAS (*P*‐value < 1 × 10^−4^). In agreement with GWAS studies for IMF in swine, our results suggest that there is a large polygenic component influencing the trait (Pena *et al.* 2016; Ros‐Freixedes *et al.* 2016; Won *et al.* 2017). However, our results also showed important genomic regions associated with IMF. Especially in OCU8, a region of 2 Mb explains a notable percentage of the genomic variance (7.34%) in comparison with other GWAS studies for IMF (Cesar *et al.* 2014; Pena *et al.* 2016).

Several genes related to lipid metabolism (on OCU1, OCU8 and OCU13) were found in the associated regions. In OCU13, orthologues of a novel gene (Ensembl gene ID: ENSOCUG00000027270) have been reported in other species. In rabbits, there are no functional annotations related to lipid metabolism or IMF linked to this gene. However, in humans and mice this gene is known as *EWS* or *EWSR1*, and regulates the genetic expression of the transcription factor ‘Y‐Box Binding Protein 1’ gene (*YBX1*). This transcription factor activates the expression of the gene *BMP7* (‘Bone Morphogenetic protein 7’), which in turn promotes the development of brown adipocytes (Wang & Seale 2016).

The genomic regions on OCU8 contained the genes with the most important biological functions. Hence, the genes on this region can be considered as candidates for further research, given that this window explains a large percentage of the IMF genomic variance (7.34%). In particular, *APOLD1*, *PLBD1*, *PDE6H* and *GPRC5A* were involved in functions of lipid transport, localisation and binding or in the control of adipose cell function. Two of these genes (*PLBD1* and *PDE6H*) participated in the catabolism of phospholipids, which are the major components of cell membranes and have important implications in adipocyte hypertrophy (Chaves *et al.* 2011; Aloulou *et al.* 2012). As a result, *PLBD1* has been related to lipid catabolic processes, skeletal muscle weight and body mass index in mice (Lionikas *et al.* 2012; Nyima *et al.* 2016) and humans (Wahl *et al.* 2017). In addition, *KRAS* (OCU8) was associated with the control of fat deposition in chickens (Claire D’Andre *et al.* 2013) and was involved in the sphingolipid signalling pathway. In humans, this gene was related to abnormal lipid metabolism in therapy for pancreatic cancer (Swierczynski *et al.* 2014). Another promising gene is *GPRC5A*, also known as *RAI3*, which is a key factor in repressing the differentiation of adipocytes in humans (Jin *et al.* 2017). This gene encodes for a member of the G‐coupled proteins, a large family including over 800 receptors, amongst them the olfactory receptors. *GRPC5A* belongs to a small subfamily of four members that are activated by retinol, the bioactive version of vitamin A. Although the role of GPRC5A is not well characterised at present, initial investigation reports a link with lung cancer, and also as a negative regulator or with adipogenesis (Song *et al.* 2019). Given the dual role of retinol during the adipogenesis (a positive regulator of pre‐adipocyte hyperplasia but a negative regulator of final maturation; see Wang *et al.* 2016a), *GRPC5A* rises as an interesting gene to mediate the inhibitory effect of retinoids in adipogenesis (Amisten *et al.* 2017).

In addition, *MTMR2* (OCU1) was linked to the metabolic process of lipids. This gene has been proposed as a functional candidate gene for IMF in GWAS and signatures of selection studies in a Duroc pig population selected for IMF (Kim *et al.* 2015).

## Conclusions and implications

This is the first GWAS study for IMF in rabbits and hence provides a benchmark for continuing research in the field. Our findings support the hypothesis that four genomic regions (on OCU1, OCU8 and OCU13) influence IMF content. The genomic variance explained by these associated regions is important although no major causal variants seem to segregate in the analysed rabbit populations. Therefore, according to what we observed in these divergently selected lines, it seems that IMF content is mainly driven by a polygenetic effect. In addition, we identified some candidate genes on the associated genomic regions of OCU13 (*EWSR1*), OCU8 (*APOLD1*, *PLBD1*, *PDE6H*, *GPRC5A* and* KRAS*) and OCU1 (*MTMR2*) related to IMF. Nevertheless, further research would be necessary in order to corroborate these results; for instance, a genotype refinement or sequencing of promoter and exonic regions of the candidate genes and its validation in independent populations of rabbits. Our results could be important for further studies to discover polymorphisms that can assist in IMF genetic improvement.

## Conflict of interests

The authors declare that there is no conflict of interests.

## Funding

The work was funded by project AGL2014‐55921‐C2‐1‐P from National Programme for Fostering Excellence in Scientific and Technical Research – Project I+D. BSS was supported by a FPI grant from the Ministry of Economy and Competitiveness of Spain+ (BES‐2015‐074194). NIB was supported with a “Ramon y Cajal” grant provided by Ministerio de Ciencia e Innovación (RYC‐2016‐19764). CSH and PN were supported by the Medical Research Council (United kingdom, grants MC_PC_U127592696 and MC_PC_U127561128). CSH was supported by Biotechnology and Biological Sciences Research Council (United Kingdom, Grant/Award Number: BBS/E/D/30002276).

## Author’s contributions

BSS carried out the statistical analyses and drafted the manuscript. AB, PH, LF and MAS conceived of the study and secured substantial funding. PH, BSS and RP performed the phenotypic data recording and collected DNA samples. PN, CSH and NIE supervised analyses and helped draft the manuscript. All authors read and approved the final manuscript.

## Supporting information


**Figure S1** LD block of the associated genomic region on OCU13.

**Figure S2 **LD block of an associated genomic region on OCU8.

**Figure S3 **Assessment of genotypes for the three relevant SNPs within genomic regions associated with intramuscular fat.

**Table S1 **Genes found in the genomic regions associated with intramuscular fat.

**Table S2 **Functions of genes identified in this study through enrichr and david.

**Table S2 **Functions of genes identified in this study through enrichr and david.

## Data Availability

The datasets used and analysed in the current study are available from the Figshare Repository (https://doi.org/10.6084/m9.figshare.9934058.v1).
