# Supplementary figures and images for: Genomic regions influencing intramuscular fat in divergently selected rabbit lines
Source: Anim Genet. 2019 Nov 7;51(1):58–69. doi: 10.1111/age.12873 (PMC7004202; doi:10.1111/age.12873)

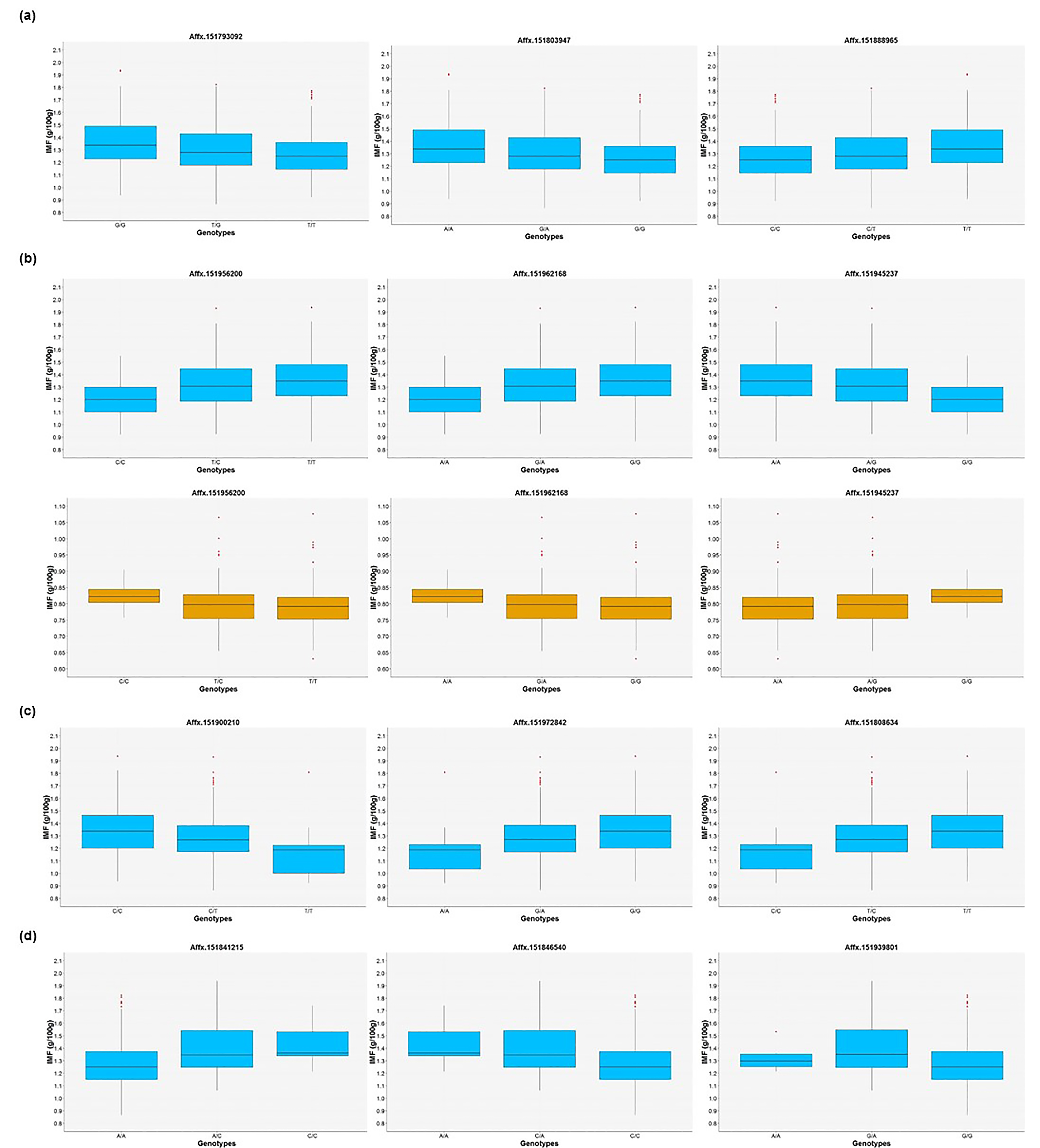

Supplement: Supplementary file 3 — Figure S3 Assessment of genotypes for the three relevant SNPs within genomic regions associated with intramuscular fat. [file AGE-51-58-s003.jpg]
