# Supplementary material for: Genomic regions influencing intramuscular fat in divergently selected rabbit lines
Source: Anim Genet. 2019 Nov 7;51(1):58–69. doi: 10.1111/age.12873 (PMC7004202; doi:10.1111/age.12873)
Supplement: Supplementary file 4 — Table S1 Genes found in the genomic regions associated with intramuscular fat. [file AGE-51-58-s004.docx]

**Additional file 4: Table S1**. Genes found in the genomic regions associated with intramuscular fat.

| **Gene stable ID** | **Rabbit gene name** | **OCU** | **Gene type** | **Gene description** |
| --- | --- | --- | --- | --- |
| **ENSOCUG00000010820** | *MAML2* | 1 | protein coding | mastermind like transcriptional coactivator 2 [Source:NCBI gene;Acc:100345650] |
| **ENSOCUG00000014568** | *MTMR2* | 1 | protein coding | myotubularin related protein 2 [Source:NCBI gene;Acc:100352381] |
| **ENSOCUG00000014557** | *CEP57* | 1 | protein coding | centrosomal protein 57 [Source:NCBI gene;Acc:100353145] |
| **ENSOCUG00000014549** | *FAM76B* | 1 | protein coding | family with sequence similarity 76 member B [Source:NCBI gene;Acc:100354406] |
| **ENSOCUG00000025632** | *SUCLA2** | 1 | protein coding | succinate-CoA ligase ADP-forming beta subunit [Source:NCBI gene;Acc:105868516 |
| **ENSOCUG00000026640** | *SESN3* | 1 | protein coding | sestrin 3 [Source:NCBI gene;Acc:100354919] |
| **ENSOCUG00000014040** | *ENDOD1* | 1 | protein coding | endonuclease domain containing 1 [Source:NCBI gene;Acc:100355178] |
| **ENSOCUG00000001631** | *KDM4D* | 1 | protein coding | lysine-specific demethylase 4D [Source:NCBI gene;Acc:100348204] |
| **ENSOCUG00000001629** | *CWC15* | 1 | protein coding | CWC15 spliceosome associated protein homolog [Source:NCBI gene;Acc:100355678] |
| **ENSOCUG00000010737** | *RASSF8* | 8 | protein coding | Ras association domain family member 8 [Source:NCBI gene;Acc:100357319] |
| **ENSOCUG00000006725** | *LMNTD1* | 8 | protein coding | lamin tail domain containing 1 [Source:NCBI gene;Acc:100357834] |
| **ENSOCUG00000028888** | *RF00001* | 8 | rRNA |  |
| **ENSOCUG00000012106** | *KRAS* | 8 | protein coding | KRAS proto-oncogene, GTPase [Source:NCBI gene;Acc:100347487] |
| **ENSOCUG00000012099** | *ETFRF1* | 8 | protein coding | electron transfer flavoprotein regulatory factor 1 [Source:NCBI gene;Acc:100347981] |
| **ENSOCUG00000015984** | *CASC1* | 8 | protein coding | cancer susceptibility 1 [Source:NCBI gene;Acc:100358611] |
| **ENSOCUG00000015956** | *LRMP* | 8 | protein coding | lymphoid restricted membrane protein [Source:NCBI gene;Acc:100358873] |
| **ENSOCUG00000003963** | *BCAT1* | 8 | protein coding | branched chain amino acid transaminase 1 [Source:NCBI gene;Acc:100337981] |
| **ENSOCUG00000021067** |  | 8 | protein coding |  |
| **ENSOCUG00000006014** | *PDE6H* | 8 | protein coding | phosphodiesterase 6H [Source:NCBI gene;Acc:100345162] |
| **ENSOCUG00000006010** | *ARHGDIB* | 8 | protein coding | Rho GDP dissociation inhibitor beta [Source:NCBI gene;Acc:100345676] |
| **ENSOCUG00000006003** | *ERP27* | 8 | protein coding | endoplasmic reticulum protein 27 [Source:NCBI gene;Acc:100347486] |
| **ENSOCUG00000016964** | *MGP* | 8 | protein coding | matrix Gla protein [Source:NCBI gene;Acc:100008989] |
| **ENSOCUG00000017191** | *ART4* | 8 | protein coding | ADP-ribosyltransferase 4 (Dombrock blood group) [Source:NCBI gene;Acc:100008862] |
| **ENSOCUG00000023339** | *SMCO3* | 8 | protein coding | single-pass membrane protein with coiled-coil domains 3 [Source:NCBI gene;Acc:100348987] |
| **ENSOCUG00000017177** |  | 8 | protein coding | WW domain binding protein 11 [Source:NCBI gene;Acc:100349748] |
| **ENSOCUG00000027663** | *H2AFJ* | 8 | protein coding | histone H2A.J [Source:NCBI gene;Acc:100350997] |
| **ENSOCUG00000017172** | *HIST4H4* | 8 | protein coding | histone H4 [Source:NCBI gene;Acc:100351746] |
| **ENSOCUG00000008162** | *GUCY2C* | 8 | protein coding | guanylate cyclase 2C [Source:NCBI gene;Acc:100008740] |
| **ENSOCUG00000026751** | *PLBD1* | 8 | protein coding | phospholipase B domain containing 1 [Source:NCBI gene;Acc:100347232] |
| **ENSOCUG00000012361** | *ATF7IP* | 8 | protein coding | activating transcription factor 7 interacting protein [Source:NCBI gene;Acc:100353017] |
| **ENSOCUG00000017095** |  | 8 | protein coding |  |
| **ENSOCUG00000021765** |  | 8 | miRNA |  |
| **ENSOCUG00000015111** | *GRIN2B* | 8 | protein coding | glutamate ionotropic receptor NMDA type subunit 2B [Source:NCBI gene;Acc:100353266] |
| **ENSOCUG00000027899** | *RF00411* | 8 | snoRNA |  |
| **ENSOCUG00000021882** |  | 8 | miRNA |  |
| **ENSOCUG00000022231** | *EMP1* | 8 | protein coding | epithelial membrane protein 1 [Source:NCBI gene;Acc:100009209] |
| **ENSOCUG00000012837** | *GSG1* | 8 | protein coding | germ cell associated 1 [Source:NCBI gene;Acc:100348479] |
| **ENSOCUG00000012826** | *FAM234B* | 8 | protein coding | family with sequence similarity 234 member B [Source:NCBI gene;Acc:100348737] |
| **ENSOCUG00000015344** | *HEBP1* | 8 | protein coding | heme binding protein 1 [Source:NCBI gene;Acc:100353525] |
| **ENSOCUG00000015331** | *GPRC5D* | 8 | protein coding | G protein-coupled receptor class C group 5 member D [Source:NCBI gene;Acc:100349237] |
| **ENSOCUG00000016556** | *GPRC5A* | 8 | protein coding | G protein-coupled receptor class C group 5 member A [Source:NCBI gene;Acc:100349493] |
| **ENSOCUG00000014390** | *DDX47* | 8 | protein coding | DEAD-box helicase 47 [Source:NCBI gene;Acc:100354028] |
| **ENSOCUG00000025481** | *APOLD1* | 8 | protein coding | apolipoprotein L domain containing 1 [Source:NCBI gene;Acc:100354429] |
| **ENSOCUG00000023584** | *RF00026* | 13 | snRNA |  |
| **ENSOCUG00000027270** | *EWSR1** | 13 | protein coding | EWS RNA binding protein 1 [Source:HGNC Symbol;Acc:HGNC:3508 |
| **ENSOCUG00000028459** | *RF00001* | 13 | rRNA |  |

* Genes annotated to the human orthologue.
