# Supplementary material for: Genomic regions influencing intramuscular fat in divergently selected rabbit lines
Source: Anim Genet. 2019 Nov 7;51(1):58–69. doi: 10.1111/age.12873 (PMC7004202; doi:10.1111/age.12873)
Supplement: Supplementary file 6 — Table S2 Functions of genes identified in this study through enrichr and david. [file AGE-51-58-s006.docx]

Figure S1. LD block of the associated genomic region on OCU13. The windows 1380 and 1381 display a shared LD block of 1506 kb. This block includes 18 SNPs of the first windows and 42 SNPs from the second window. The red colour indicates a high LD and the blue color indicate a low LD.

Figure S2. LD block of an associated genomic region on OCU8. The windows 841 and 842 display a shared LD block of 1945 kb. The block includes 145 SNPs. The red colour indicates a high LD.

Figure S3. Assessment of genotypes for the three relevant SNPs within genomic regions associated with intramuscular fat. The light blue colour denotes high-IMF line and orange colour denotes low-IMF line. Boxplots of polymorphisms (SNPs) in (a) OCU1 (120.65–121.99 Mb), (b) OCU8 (14.01–15.47 Mb), (c) OCU8 (24.59–26.95 Mb), and (d) OCU13 (83.81–86.00 Mb). The SNPs in the regions (a), (c), and (d) displayed minor allele frequencies (MAF) below 0.03 (close to zero) within low-IMF line precluding their assessment
